# Re-sequencing of mitochondrial genes in a standard rice cultivar Nipponbare

**DOI:** 10.1186/1939-8433-6-2

**Published:** 2013-01-10

**Authors:** Takushi Toda, Kinya Toriyama

**Affiliations:** Graduate School of Agricultural Science, Tohoku University, Sendai, 981-8555 Japan

**Keywords:** Rice, Mitochondria, Re-sequencing, SNP

## Abstract

**Background:**

Genomic sequence of a rice cultivar Nipponbare has been often used as a reference sequence since a whole-genomic sequence was first determined in 2005 by the International Rice Genome Sequencing Project. As for mitochondrial genomic sequence of Nipponbare, two groups have deposited their sequences into DDBJ/EMBL/GenBank under the accession numbers BA000029 and DQ167400. However, there are 19 discrepancies in the nucleotide sequences of 7 genes between BA000029 and DQ167400.

**Findings:**

We performed PCR to amplify these 7 genes and to perform direct sequencing. Nucleotides of the discrepant sites were all identical to those in DQ167400.1. The sequence in BA000029.3 is thought to contain sequencing errors.

**Conclusion:**

Nucleotide sequences of the mitochondrial genes in BA000029.3 need to be updated using the data in this study when used as a reference genome.

**Electronic supplementary material:**

The online version of this article (doi:10.1186/1939-8433-6-2) contains supplementary material, which is available to authorized users.

## Findings

The International Rice Genome Sequencing Project adopted a *japonica* rice cultivar Nipponbare as a standard and determined its whole-genomic sequences (International Rice Genome Sequencing Project, 2005). Mitochondrial genome of Nipponbare has been sequenced by two groups. Notsu et al. (2002) used plasmid clones containing the “shotgun” DNA fragments derived from isolated mitochondrial DNA for sequence analysis employing a conventional chain terminating method. The sequence is available in DDBJ/EMBL/GenBank under the accession number BA000029.3. Tian et al. (2006) extracted the mitochondrial sequences from a whole-genome shotgun nuclear sequence, in which sequencing was done with MegaBACE 1000 capillary sequencers employing a conventional chain terminating method. The sequence is available in DDBJ/EMBL/GenBank under the accession number DQ167400.1. These Nipponbare sequences are often used as reference genomes for the studies of cytoplasmic male sterility (CMS) and C-U RNA editing (Fujii et al. 2010; Bentolila and Stefanov 2012; Toda et al. 2012).

First, we compared the sequences of 35 known protein-coding genes, three ribosomal RNAs, and 17 tRNA genes between BA000029.3 and DQ167400.1. We found 19 discrepancies among nucleotides in 7 genes; *ccmFn* (cytochrome c biogenesis Fn), *mat-r* (maturase-related protein), *nad4* (NADH dehydrogenase subunit 4), *nad6* (NADH dehydrogenase subunit 6), *rpl2* (ribosomal protein L2), *rrn18* (18S ribosomal RNA) and *rrn26* (26S ribosomal RNA) (Table 1). Some of the nucleotide differences predicted amino acid changes, which were unlikely to happen within the same cultivar Nipponbare.

**Table 1:** **Comparison of SNP among mitochondrial sequences reported by Notsu et al. (** 2002 **), Tian et al. (** 2006 **) and the current study**

Gene	CDS Length (bp)	Position of SNP	Position of SNP in BA000029.3	Notsu et al. (2002)	Tian et al. (2006)	Current study	
				BA000029.3	DQ167400.1		
				genomic seq	genomic seq	genomic seq	cDNA seq
*ccmFn*	1,785	185	323,671	gGt (G)	gCt (A)	gCt (A)	gCt (A)
		677	323,179	gCc (A)	gGc (G)	gGc (G)	gGc (G)
*mat-r*	2,037	1,809	316,040	cgT (R)	cgA (R)	cgA (R)	cgA (R)
		1,812	316,037	acC (T)	acG (T)	acG (T)	acG (T)
*nad4*	1,488	759	200,397	gcT (A)	gcA (A)	gcA (A)	gcA (A)
*nad6*	618	21	60,116	tcT (S)	tcG (S)	tcG (S)	tcG (S)
		97	60,040	Atc (I)	Gtc (V)	Gtc (V)	Gtc (V)
		166	59,971	Att (I)	Gtt (V)	Gtt (V)	Gtt (V)
		398	59,739	aAt (N)	aGt (S)	aGt (S)	aGt (S)
		414	59,723	gaC (D)	gaA (E)	gaA (E)	gaA (E)
		467	59,670	aCt (T)	aGt (S)	aGt (S)	aGt (S)
		474	59,663	atC (I)	atT (I)	atT (I)	atT (I)
		476	59,661	tCa (S)	tTa (L)	tTa (L)	tTa (L)
		562	59,575	Ctg (L)	Ttg (L)	Ttg (L)	Ttg (L)
		567	59,570	gaC (D)	gaT (D)	gaT (D)	gaT (D)
		607	59,530	Tcg (S)	Acg (T)	Acg (T)	Acg (T)
*rpl2*	1,509	1,249	297,421	Ttc (F)	Ctc (L)	Ctc (L)	Ctc (L)
*rrn18*	1,695	1,566	282,896	C	T	T	T
*rrn26*	3,542	1,980	382,078	G	C	C	C

The position of the SNP is shown relative to the start site of each protein-coding gene or mature rRNA. Each SNP is capitalized in the corresponding codon, with the amino acid change shown in parentheses.

To re-sequence the above mentioned genes and their cDNAs of Nipponbare, genomic DNA and total RNA were isolated using the DNeasy Plant Mini kit (QIAGEN, Tokyo, Japan) and RNAiso (TaKaRa Bio, Otsu, Japan), respectively, from 7-week-old seedlings. Ten μg of RNA was treated with DNase I (TaKaRa Bio, Otsu, Japan), and the resulting RNA was reverse-transcribed with Rever Tra Ace (TOYOBO, Osaka, Japan) using random primers (TaKaRa Bio, Otsu, Japan). The genomic and cDNA fragments were amplified by 40 cycles of a standard PCR using Ex-taq polymerase (TaKaRa Bio, Otsu, Japan) and purified using the UltraClean® 15 DNA Purification Kit (MO BIO Laboratories, Inc., CA, USA). Direct-sequencing of the PCR products was performed using the CEQ8000 Genetic analysis system (Beckman Coulter, Fullerton, California). Primers used for PCR and sequencing are listed in Table 2.

**Table 2:** **Primers using direct sequence analysis**

Mitochondrial genes	Sequence
*ccmFn* (185)	5^′^-GCAGAAATTTTGAAATGAGC-3^′^
	5^′^-GCCCCACTTTTTTGTTGGAG-3^′^
*ccmFn* (677)	5^′^-GTTACATCTGGCACGAGATG-3^′^
	5^′^-GTGCCACAATCCCATTCATC-3^′^
*mat-r*	5^′^-GCGAATTCCCCATACAGATA-3^′^
	5^′^-TCCGTCGTTGTTCGGATCTT-3^′^
*nad4*	5^′^-GGATTGCTTTTTTCGCCTCT-3^′^
	5^′^-CTGTCTTAAAGTGGTCAAGG-3^′^
*nad6*	5^′^-TTCCATTTCAAGGGAGGACG-3^′^
	5^′^-ACGTGGACTCGAACCACAAT-3^′^
*rpl2*	5^′^-ATCCAGGTCAAGGCGCAAAG-3^′^
	5^′^-TTTCTAAGCTTACGTGCACC-3^′^
*rrn18*	5^′^-CGTGCTACAATGGCAATGAC-3^′^
	5^′^-CTTGTTACGACTTCACCCCA-3^′^
*rrn26*	5^′^-ACTGGCGCTTCCAAAGGCGA-3^′^
	5^′^-CACACGTGAAAGTGCTTCGA-3^′^

As shown in Table 1 and in Additional file 1, nucleotides of the discrepant sites in the genomic sequences were all identical to those in DQ167400.1. In addition, cDNA sequences were also identical to the genomic sequences in DQ167400.1 (Table 1). Thus, BA000029.3 is considered to contain sequencing errors. We also compared these nucleotides among mitochondrial genomes of other cultivars and wild rice in databases; a cultivar PA64S with *japonica*-derived mitochondria (DQ167807.1; Tian et al. 2006), an *indica* cultivar 93–11 (DQ167399.1; Tian et al. 2006), IR6888 (JF281153.1; Bentolila and Stefanov 2012), and Lead rice (AP011077.1; Fujii et al. 2010), and cultivars carrying wild rice (*Oryza rufipogon*) mitochondria, WA-CMS line (JF281154.1; Bentolila and Stefanov 2012) and CW-CMS line (AP011076.1; Fujii et al. 2010). Among all the above mentioned mitochondrial genomes, the genes shown in Table 1 show consistent sequences to those in DQ167400.1.

Bentolila and Stefanov (2012) have reported complete sequences of *indica* cultivar IR6888 and WA-CMS mitochondrial genomes and presented a table of substitutions and insertions within rice mitochondrial genes relative to Nipponbare using BA000029.3 as a reference genome. They claimed that they found 26 single-nucleotide polymorphisms and stated that it was unclear why *nad6* in Nipponbare exhibited many polymorphisms relative to *nad6* genes in the other rice mitochondrial genomes. Our current study indicated that the sequencing errors in BA000029.3 led to a different conclusion. The sequencing errors in BA000029.3 should be revised. The position of the nucleotide that should be updated in BA000029.3 is indicated in Table 1. Meanwhile the nucleotide sequence in DQ167400.1 should be used as a reference sequence for mitochondrial genes of Nipponbare.

## Accession codes

Nucleotide sequence data obtained in our study have been deposited to the DDBJ under accession numbers [AB763976 to AB763991].

## Authors' information

Laboratory of Environmental Plant Biotechnology, Graduate School of Agricultural Science, Tohoku University, Sendai 981–8555, Japan.

## Electronic supplementary material

Additional file 1:**Sequencing chromatograms derived from direct sequencing of the genomic PCR products of the genes listed in Table**1**.** The position of the SNP is shown relative to the start site of each protein-coding gene or mature rRNA. (PDF 76 KB)

## Abbreviations

CMS: Cytoplasmic male sterility
SNP: Single nucleotide polymorphism.

## References

[CR1] Bentolila S, Stefanov S (2012). A reevaluation of rice mitochondrial evolution based on the complete sequence of male-fertile and male-sterile mitochondrial genomes. Plant Physiol.

[CR2] Fujii S, Kazama T, Yamada M, Toriyama K (2010). Discovery of global genomic re-organization based on comparison of two newly sequenced rice mitochondrial genomes with cytoplasmic male sterility-related genes. BMC Genomics.

[CR3] International Rice Genome Sequencing Project (2005). The map-based sequence of the rice genome. Nature.

[CR4] Notsu Y, Masood S, Nishikawa T, Kubo N, Akiduki G, Nakazono M, Hirai A, Kadowaki K (2002). The complete sequence of the rice (Oryza sativa L.) mitochondrial genome: frequent DNA sequence acquisition and loss during the evolution of flowering plants. Mol Genet Genomic.

[CR5] Tian XJ, Zheng J, Hu SN, Yu J (2006). The rice mitochondrial genomes and their variations. Plant Physiol.

[CR6] Toda T, Fujii S, Noguchi K, Kazama T, Toriyama K (2012). Rice MPR25 encodes a pentatricopeptide repeat protein and is essential for RNA editing of nad5 transcripts in mitochondria. Plant J.

