# Supplementary figures and images for: Re-sequencing of mitochondrial genes in a standard rice cultivar Nipponbare
Source: Rice (N Y). 2013 Jan 10;6:2. doi: 10.1186/1939-8433-6-2 (PMC5394982; doi:10.1186/1939-8433-6-2)

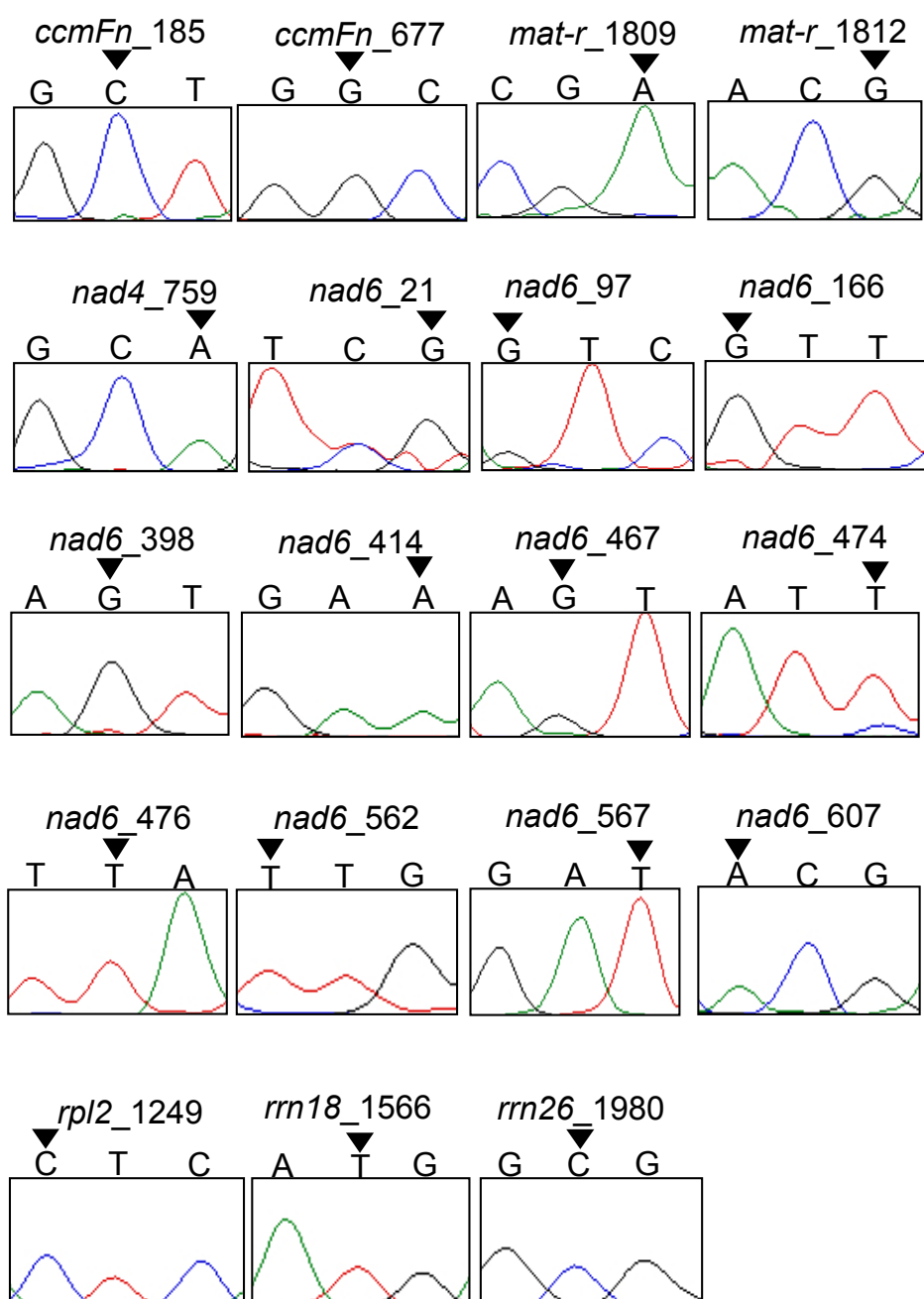

Supplement: Supplementary file 1 — Additional file 1:Sequencing chromatograms derived from direct sequencing of the genomic PCR products of the genes listed in Table1. The position of the SNP is shown relative to the start site of each protein-coding gene or mature rRNA. (PDF 76 KB) [file 12284_2012_40_MOESM1_ESM.pdf]
